# Hearing Loss and Healthcare Satisfaction

**DOI:** 10.1093/geroni/igaa057.691

**Published:** 2020-12-16

**Authors:** Anna Jilla, Nicholas Reed

**Affiliations:** Johns Hopkins University, Baltimore, Maryland, United States

## Abstract

Patient satisfaction with care has become increasingly important since Medicare’s shift to value-based reimbursement models using the Hospital Care Quality Information from the Consumer Perspective survey. Effective communication plays an underappreciated role in satisfaction with care. Hearing loss impacts two-thirds of adults over 70 years and is a barrier to communication. Previous research has found that adults with hearing loss have poorer health outcomes and incur higher medical expenditures. The present study aims to explore the association of hearing loss on satisfaction with care. Nationally representative data from the 2015 Medicare Current Beneficiary Survey (MCBS) collected information on self-reported hearing with a hearing aid (no trouble, a little trouble, or a lot of trouble) and satisfaction with quality of health care (very satisfied/satisfied and dissatisfied/very dissatisfied) for all adult Medicare beneficiaries. A weighted sample of 48.6 million Medicare Beneficiaries was analyzed using logistic regression, adjusted for sex, race, educational attainment, income, general health, and functional limitations in instrumental activities of daily living. The adjusted model found that compared to adults with no hearing trouble, those with a little trouble hearing and a lot of trouble hearing had 1.47 times (95% CI: 1.06, 2.03) and 1.74 times higher odds (95% CI: 1.15, 2.62) higher odds of reporting dissatisfaction with care, respectively. Hearing loss, possibly mediated by its impact on communication, is associated with satisfaction with care. Given the emphasis placed on patient-reported satisfaction value-based reimbursement programs, hearing loss may represent a potential, low-risk/high-reward area of intervention to improve satisfaction.

